# High Resolution Dissection of Reactive Glial Nets in Alzheimer’s Disease

**DOI:** 10.1038/srep24544

**Published:** 2016-04-19

**Authors:** David S. Bouvier, Emma V. Jones, Gaël Quesseveur, Maria Antonietta Davoli, Tiago A. Ferreira, Rémi Quirion, Naguib Mechawar, Keith K. Murai

**Affiliations:** 1 Centre for Research in Neuroscience, Department of Neurology and Neurosurgery, The Research Institute of the McGill University Health Centre, Montreal General Hospital, Montreal, Quebec, Canada; 2 Douglas Mental Health University Institute, Department of Psychiatry, McGill Group for Suicide Studies, McGill University, Montreal, Quebec, Canada

## Abstract

Fixed human brain samples in tissue repositories hold great potential for unlocking complexities of the brain and its alteration with disease. However, current methodology for simultaneously resolving complex three-dimensional (3D) cellular anatomy and organization, as well as, intricate details of human brain cells in tissue has been limited due to weak labeling characteristics of the tissue and high background levels. To expose the potential of these samples, we developed a method to overcome these major limitations. This approach offers an unprecedented view of cytoarchitecture and subcellular detail of human brain cells, from cellular networks to individual synapses. Applying the method to AD samples, we expose complex features of microglial cells and astrocytes in the disease. Through this methodology, we show that these cells form specialized 3D structures in AD that we refer to as reactive glial nets (RGNs). RGNs are areas of concentrated neuronal injury, inflammation, and tauopathy and display unique features around β-amyloid plaque types. RGNs have conserved properties in an AD mouse model and display a developmental pattern coinciding with the progressive accumulation of neuropathology. The method provided here will help reveal novel features of the healthy and diseased human brain, and aid experimental design in translational brain research.

With an aging population worldwide and increasing number of individuals developing neurodegenerative diseases, new methods that better decipher the cellular properties of the human brain are needed. Light microscopic analysis of post-mortem brain tissue remains an important method for revealing features of normal and diseased brain cells. However, significant obstacles with this approach still prevent the acquisition of high-quality three-dimensional (3D) information about cells from samples obtained from long-term storage. Strong tissue auto-fluorescence, poor antibody penetration, and non-uniform labeling are major limitations that commonly restrict imaging and analysis to thin (5–10 μm), slide-mounted tissue sections. Recent tissue ‘clearing’ methods have improved light penetration in thick samples allowing a better overview of cellular organization and have shown some compatibility with human tissue^1^,^2^. However, these techniques involve extensive tissue processing steps and can cause distortion of normal tissue dimensions/properties^1^,^2^,^3^. Thus, a robust and broadly accessible method that preserves cellular architecture and enables the dissection of 3D properties of cells in the human brain is needed. This type of method would be especially beneficial for understanding complex brain diseases such as Alzheimer’s disease (AD), which involve simultaneous pathological events including neuronal dysmorphism, β-amyloid_1−42_ (now referred to as Aβ) plaque deposition, neurofibrillary tangle accumulation, hyper-phosphorylation of tau, as well as, glial reactivity and neuroinflammation^4^. Parsing how these cellular and molecular changes are temporally and spatially coordinated in AD will allow stronger hypotheses to be developed and tested.

Recently, glial reactivity and neuroinflammation have received significant attention because of their potential to exacerbate or inhibit Aβ and tau-related AD pathologies^5^. Activated microglia and reactive astrocytes have been observed surrounding Aβ plaques in human AD brain tissue since the studies of Alois Alzheimer^6^, and have been described near amyloid inclusions by electron and light microscopy^7^,^8^,^9^. However, the precise role of microglia and astrocytes is still actively debated, with both positive and negative roles attributed to these cells in AD^10^,^11^. Fueling the debate is the fact that microglia and astrocytes adopt heterogeneous molecular properties in response to CNS injury and disease^12^,^13^,^14^,^15^. The presence of microglia and astrocytes with distinct anatomical, reactive, and inflammatory profiles could drastically influence the fate of surrounding neurons. Thus, greater ability to resolve the *in situ* subcellular details of these cells, as well as, their 3D organization will help in determining the function of these cells in AD.

Here we developed a robust and reliable method that allows multi-channel, high-resolution 3D light microscopic analysis of human brain tissue stored up to 25 years in fixative. Importantly, this method provides simultaneous resolution of 3D relationships between neurons, microglia, and astrocytes across large tissue landscapes and within thick human specimens, as well as, detailed subcellular localization of proteins within these cells. Applying the method, we uncovered specific 3D microglia-astrocyte structures around Aβ plaques in cerebral cortex of AD brain that we refer to as reactive glial nets (RGNs). RGNs are areas of concentrated inflammation, neuronal injury, and tauopathy and display unique structural and molecular features according to Aβ plaque type, thus enabling sub-classification of different types of Aβ pathology in the disease. Using information gained from human tissue analysis, we demonstrated that RGNs have conserved features in an AD mouse model and a progressive development of inflammation that coincides with local accumulation of neuropathology.

## Results

### A broadly-accessible and robust method for resolving neurons and glial cells in long-term fixed human brain

We developed a method to improve the efficiency, reliability, and quality of labeling in human brain samples stored for years in fixative. Important in the method is the flexibility to perform multi-labeling of thick, free-floating tissue sections and high-resolution, 3D light microscopic analysis without the need for complex reagents and tissue processing steps. Fixed samples of human temporal and frontal cortices were cryoprotected, embedded, and cut to produce 50 μm free-floating sections (Suppl. Fig. 1a). To dampen the strong auto-fluorescence of fixed human tissue, we exposed free-floating sections to ultraviolet light for 18–24 hrs prior to antibody labeling. This step significantly cut autofluorescence and enhanced signal-to-noise detection (Suppl. Fig. 1b)^16^. We next developed a procedure for deep, uniform antibody penetration in sections, while minimizing unspecific crosstalk between labels. The sequential replenishment with fresh primary antibody solution was critical for samples with low antigenicity or poor antibody penetration and greatly improved the quality of the labeling in x, y and z-axes (Suppl. Fig. 1c). Successive rounds of primary and secondary antibodies also enabled deeper penetration (up to 100 μm in the z-axis) of multiple labels into thick tissue slices, allowing large volume reconstructions (Suppl. Fig. 1d), and avoided the uneven, superficial layering of the antibodies often observed with this type of procedure. High-quality labeling was achieved independent of age, brain region (temporal and frontal cortex; hippocampus (Suppl. Fig. 2), status of brain tissue (control and AD), type of fixative (paraformaldehyde or formalin), or post-mortem interval (5.6–35.5 hours). 10 of 10 different human samples processed with this approach (average storage 15.3 years), including a sample preserved in fixative for 25 years, were effectively immunolabeled with this method.

**Figure 1:**
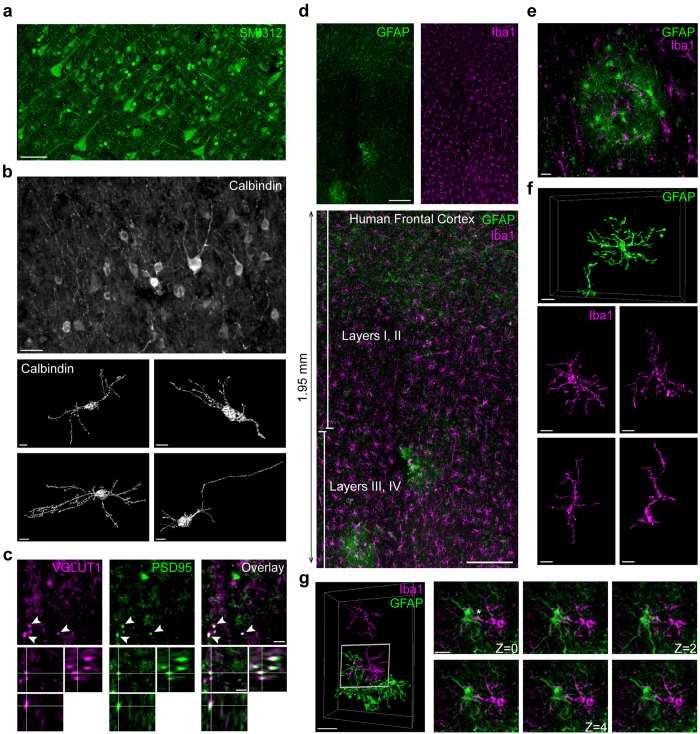
A broadly accessible, systematic method to study neurons and glia in human brain samples recovered from long-term storage. (**a**) The staining of pan-axonal neurofilaments (SMI312) reveals the general organization of neurons in cortical sections (female, 86 years old, 25 years of fixation). (**b**) Calbindin expressing interneurons are enriched in layers I/II of the human cortex (male, 88 years old, 19 years of fixation) and show diversified morphologies after 3D reconstruction. (**c**) High-resolution imaging of synapses in cortex (female, 82 years old, 15 years of fixation) shows vGlut1-positive presynaptic boutons (magenta) juxtaposed to PSD95-positive postsynaptic structures (green) (synapses indicated by arrows). (**d**) Maximum projection of a large field showing 1,95 mm^2^ (x:1.95 mm, y:0.9 mm) of tissue with a 30 μm depth labeled for Iba1 (microglia; magenta) and GFAP (astrocytes; green). (**e**) High magnification image of a GFAP-positive astrocyte ‘island’ in a cortical section. (**f**) 3-dimensional reconstructions of cortical protoplasmic GFAP+ astrocytes and Iba1+ microglia (female, 86 years old). (**g**) GFAP+ astrocytes (green) and Iba1+ microglia (magenta) in cortical layers III/IV show complex interactions in a control brain (female, 86 years old), resolvable by visualizing individual frames of confocal Z-stacks (step-size: 1 μm). Scale bars: 50 μm (**a**); 20 μm (**b**); 5 μm (**c**); 200 μm (**d**); 10 μm (**e**–**g**).

To demonstrate the utility of this method, we analyzed the organization and morphology of brain cells in cortical samples from healthy, aged individuals (average age, 84.2 years; average storage, 20.4 years; Table 1). Specific antibodies were used to label neurons (SMI312, calbindin), microglia (ionized calcium-binding adapter molecule 1; Iba1), and astrocytes (Glial Fibrillary Acidic Protein; GFAP). Staining of neurofilaments (SMI312) resolved the layered organization of neurons in neocortex and their general morphology (Fig. 1a). Discrete populations of pyramidal neurons including calbindin D28-positive interneurons of layer III were detected, allowing their complex morphology to be visualized (Fig. 1b). Labeling for the presynaptic protein VGLUT1 and postsynaptic scaffold PSD95 demonstrated the ability of this approach to resolve individual synapses (Fig. 1c). Thus, this method allows for simultaneous analysis of neuronal populations, single-cell anatomy, and synaptic organization in long-term fixed human brain tissue.

We next applied this methodology to two glial cell types in the brain, microglia and astrocytes. Microglia are the primary immune cells in the brain that survey the extracellular milieu for foreign antigens and play a central role in nervous system inflammation^13^,^17^. In contrast, astrocytes extend elaborate processes between neurons, blood vessels, and synapses and function in brain homeostasis and synaptic plasticity^15^. As these glial cell types form large cellular networks throughout brain tissue, we constructed large landscapes (mm^2^) of human frontal cortex using the approach. This showed a remarkable distribution of astrocytes and microglia (Fig. 1d). Labeling of GFAP+ astrocytes was heterogeneous in intensity and organization, revealing a distinct astrocyte population. Strongly labeled interlaminar GFAP+ astrocytes with long processes and varicosities were especially prevalent in layers I/II, while weakly labeled protoplasmic astrocytes populated layers III–VI (Fig. 1d–f). These features of GFAP+ astrocytes have previously been described in detail in surgically resected, acutely fixed tissues from patients with pathological conditions^18^. We also observed intriguing astrocyte “islands” with GFAP+ cells clustered within cortical layers III–VI (Fig. 1e).

Much less is known about the characteristics of human microglia^19^,^20^,^21^. We found Iba1+ microglia to be uniformly distributed in frontal and temporal cortices (Fig. 1d). High-magnification imaging revealed many different microglial cell morphologies with some cells showing round-shaped cell bodies with ramified processes to some microglia exhibiting elongated somas with main processes that were poorly elaborated (Fig. 1f). Heterogeneity of microglia morphology may represent different states of surveillance or “activation” of microglia in the aging brain as reported in autopsy tissue^20^. Interestingly, microglia and astrocytes showed complex 3D structural interactions that were resolved in successive confocal image stacks (Fig. 1g). Thus, the described approach allows a clear view of cytoarchitecture and subcellular anatomy within long-term fixed human tissue.

### High-resolution analysis of the pathohistological hallmarks of AD

**Figure 2:**
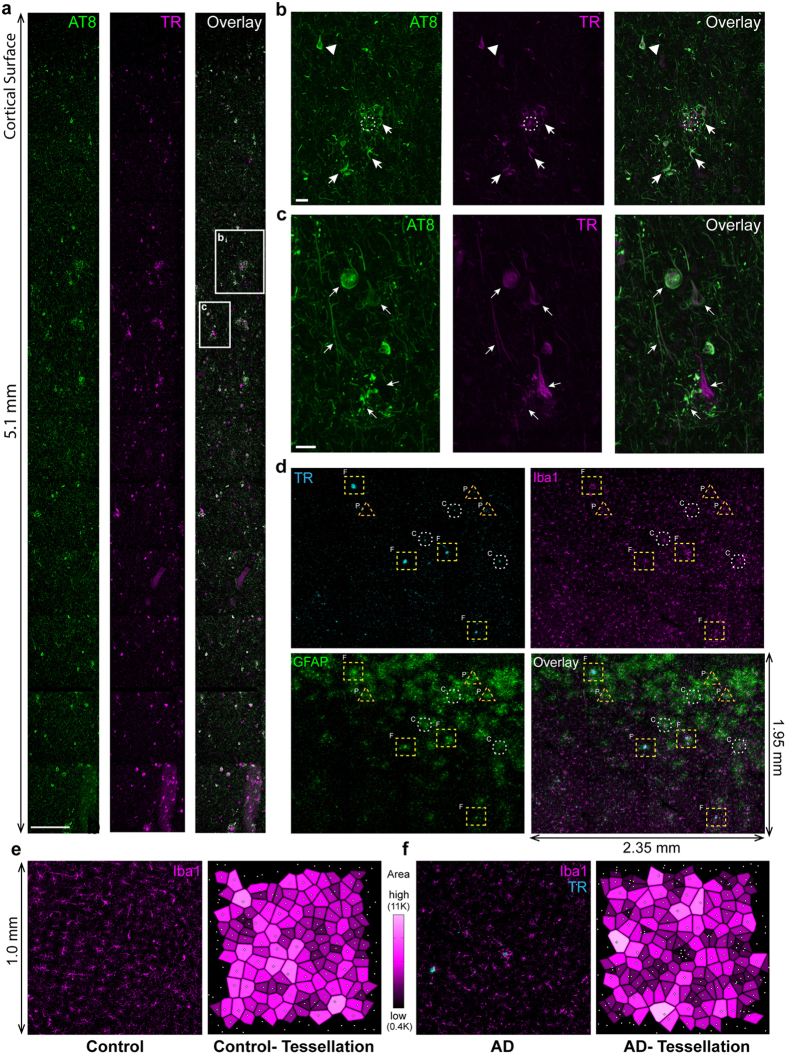
Exposing macro- and microscopic AD pathology in post-mortem brain samples. (**a**) Maximum projection of a vertical column of cortical tissue (5.1 mm X 0.468 mm and 40 μm thick) from an AD patient brain samples (female, 85 years old) and labeled with an AT8 antibody (Phospho-PHF-tau pSer202+Thr205) and Thiazine-red (TR) that reveal Aβ plaques, PHF, and NFTs. (**b**,**c**) Individual maximum projections of image fields from panel (**a**). (**d**) Maximum projection showing a 4.6 mm^2^ (x: 2.35 mm, y: 1.95 mm) of temporal cortex from an AD sample (male, 87 years old) with a 30 μm imaging depth and labeled for Iba1 (microglia; magenta), GFAP (astrocytes; green), and Thiazine-Red (plaques, cyan). TR staining reveals the presence of subgroups of plaques/aggregates. The dense-core Aβ plaques (C, dashed white hexagons) are the most numerous while larger fibrillar amyloid plaques (F, dashed yellow squares) are found in older patients. PHF aggregates are also frequently observed (P, dashed orange triangles). Iba1+ cell clusters are detected around dense-core and fibrillar plaques but are absent around PHF aggregates. Coronas of reactive astrocytes are observed around the 3 types of plaques/aggregates. (**e**,**f**) General distribution of Iba1+ microglia in control and AD brain tissue (1 mm^2^). Topological density of Iba1-positive microglia before and after Voronoi segmentation, in which individual microglia territories are color-coded according to their surface area. The homogeneity of microglial cell distribution that we have measured in the healthy brain (left) is disrupted by the presence of plaques (right) in AD brain with individual microglial territories becoming smaller near plaques (darker areas) and larger in adjacent areas (lighter areas), suggest microglial cell aggregation near deposits and their depletion from adjacent areas. Scale bars: 200 µm (**a**) 20 µm (**b,c**).

The anatomical distribution of Aβ plaques and neurofibrillary tangles (NFTs) through the course of AD has been characterized in human AD post-mortem samples two decades ago^22^,^23^. Aβ plaques are commonly categorized according their morphology and the presence of surrounding abnormal neuronal structures^24^. Thus, Aβ plaques are commonly divided into two sub-groups, dense-core and diffuse plaques, with dense-core plaques commonly associated with surrounding dysmorphic glial cells and neurons/neurites^24^. However there is a need to update these classifications that were originally based upon traditional immunohistochemistry techniques that do not provide sufficient resolution to detect more subtle alterations in the spatial organisation of neuronal and glial cells in AD brain. Although the mechanisms underlying the neurodegenerative events in AD remain to be fully understood, the disease is recognized as a multifactorial disorder^4^. Aβ plaques, NFTs, glial reactivity, and inflammation are all signatures of the disease. However, exactly how these pathological features are spatially coordinated in the AD brain requires further investigation. With a cohort of AD samples (Table 1), we applied the labeling/imaging method to resolve cellular and subcellular changes in AD. We applied Thiazine Red (TR) labelling which is commonly used on post-mortem samples for diagnostic purposes^25^ to reliably mark dense-core Aβ plaques and tangles. Importantly, TR is also compatible with multi-antibody labeling procedures, penetrating deep into thick brain tissue (Suppl. Figs 1 and 2). While large 3D image landscapes showed the overall organization Aβ plaques, aggregates of paired-helical filaments (PHFs), and NFTs^23^,^24^ (Fig. 2a), individual image stacks embedded within these landscapes unveiled the detailed 3D pathology of degenerating neurons (Fig. 2b,c). To understand microglial-astrocyte relationships with pathological hallmarks of AD, we labeled for Iba1+ microglia and GFAP+ astrocytes and performed 3D imaging of millimeter-size territories of frontal cortex. This revealed a striking arrangement of astrocytes and microglia near large Aβ deposits and arrays of PHFs (Fig. 2d). GFAP+ astrocytes densely populated areas of high Aβ plaque load and NFT density. Iba1+ microglia also showed an altered distribution, albeit in a different manner, than astrocytes. Maximum projections of image stacks enabled Voronoi tessellation and nearest-neighbor distance analysis of microglia, revealing their irregular distribution in AD with microglial aggregation near plaques and depletion in adjacent zones (Fig. 2e,f; Suppl. Fig. 3d,e).

We encountered three distinct types of AD pathology labeled by TR (Fig. 2d, Suppl. Fig. 3a–c). Dense-core plaques usually referred to as “classical” plaques^26^, showed a core of Aβ often surrounded by additional TR-labeled fibrils^25^,^27^ (Fig. 2d, Suppl. Fig. 3a; hexagons). These plaques were numerous and found in five-of-five AD samples. However a second type of TR+ plaque with distinct morphological features was detected in three-of-five AD samples (DH-1073, 1157 and 1631). These plaques were previously referred as “fibrillar”^28^,^29^ because their lack of a prominent core and the presence of condensed Aβ fibrils (Fig. 2d, Suppl. Fig. 3b; squares). Both dense-core and fibrillar plaque types were mainly found within layer IV-VI of cortical tissue, surrounded by abnormal neurites (SMI 312 labeled, Suppl. Fig. 3a,b) and clustered microglia and astrocytes. In addition to these plaque types, we observed paired helical filaments (PHFs) and/or NFTs, co-labeled with phosphorylated forms of tau (AT8 and PS422) clustered in dense spots devoid of Aβ core and fibrillar structures that were especially prevalent in the first four layers of the cortex that we refer to as PHF/NFT aggregates (Fig. 2d, Suppl. Fig. 3c; triangles). Diffuse amyloid plaques were not stained by TR. TR+ plaques were only found in 1 of 5 control samples, with the one patient having notable arteriosclerosis. Interestingly, dense-core and fibrillar Aβ deposits in AD samples were all associated with microglia-astrocyte accumulation as revealed through the presence of robust IBA+ microglia and GFAP+ astrocytes (Fig. 2d).

### Distinct features of microglia-astrocyte associations with Aβ plaque types

**Figure 3:**
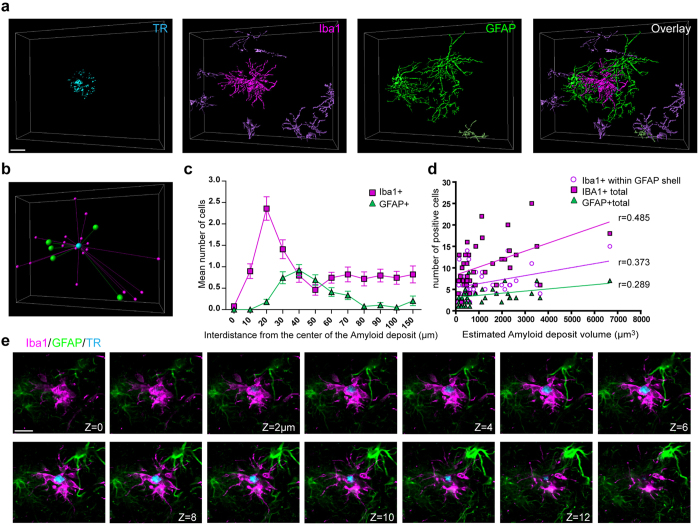
Specialized Reactive Glial Nets (RGNs) form around dense-core Aβ plaques in AD cortex. (**a**) 3-dimensional reconstruction of a confocal Z-stack showing GFAP+ reactive astrocytes (green) and Iba1+ microglia (magenta) surrounding a Thiazine red-labeled dense-core Aβ plaque (cyan) in an AD patient (female, 77 year old). Note the TR-labeled fibrils surrounding the core of the plaque. (**b**) 3D analysis of astrocyte and microglia position around a dense-core plaque. (**c**,**d**) Quantification of the positioning of astrocytes and microglia relative to plaques and the positive correlation between astrocyte and microglial cell number and plaque size (n = 39). (**e**) Sequence of 14 successive z- focal planes (1 μm step size) showing amoeboid Iba1+ cells (magenta) enveloping the core of the plaque (cyan) and reactive astrocytes circumscribing them with processes. Note GFAP+ astrocytic processes are excluded from the microglial cell territory. Scale bars: 20 μm.

**Figure 4:**
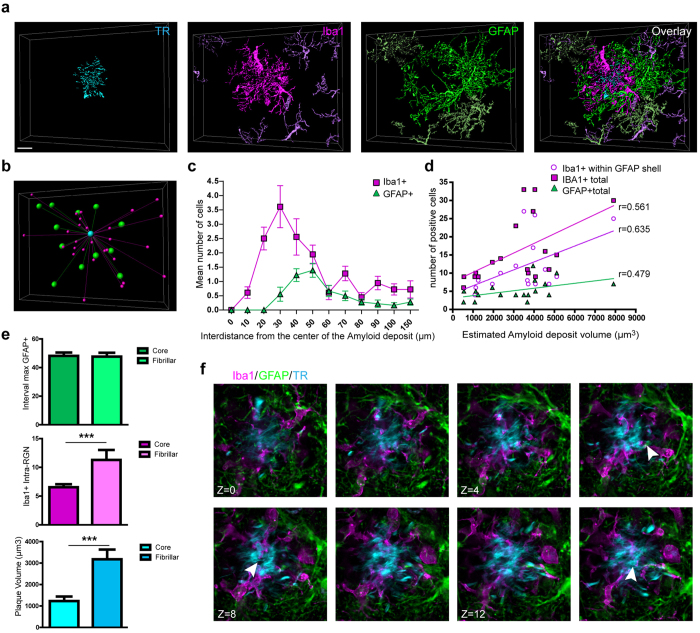
RGNs surrounding fibrillar Aβ plaques. (**a**) 3D reconstruction of a confocal Z-stack showing GFAP+ reactive astrocytes (green) and Iba1+ microglia (magenta) surrounding Thiazine red-labeled fibrillar Aβ plaque (cyan) in a AD patient (male, 87 years old). (**b**) 3D analysis of astrocyte and microglia position around a fibrillar plaque. (**c**,**d**) Quantification of the positioning of astrocytes and microglia relative to plaques and the positive correlation between astrocyte and microglial cell number and plaque size. (n +18; r = 0.561 for total Iba1+ cells, r = 0.635 for Iba1+ cells within GFAP shell, and r = 0.479 for GFAP+ cells) (**e**) Comparison of dense-core and fibrillar plaque subtypes of interval of inter-distance of GFAP+ cells from plaques (F(1, 55) = 0.016; p = 0.8894), numbers of Iba1+ cells within the astrocyte shell of RGNs (F(1, 55) = 11.276; p < 0.001), and volume of the plaques (F(1, 55) = 19.841; p < 0.0001). ***p < 0.001: significantly different from dense-core plaque. (**f**) Sequence of 8 successive focal planes (2 μm step size) showing Iba1+ microglial (magenta) and GFAP+ astrocytes (green) with astrocytic processes (arrowheads) invading the Aβ fibrillar masse (cyan). Scale bars: 20 μm.

Microglia and astrocytes are tightly interleaved among neurons in the healthy brain but undergo extensive structural and molecular changes in AD. However, the role of glial cells and their reorganization in AD is still actively debated^5^. This is due, in part, to the heterogeneity of their morphology and molecular profile^10^,^17^,^30^,^31^,^32^. To decipher if microglia and astrocytes exhibit specific features around dense-core and fibrillar Aβ deposits and PHF/NFT aggregates, we performed detailed 3D analysis of their spatial organization. This revealed an elaborate 3D glial structure, with an inner sphere of dysmorphic/amoeboid microglia and an outer sphere of hypertrophic astrocytes around dense-core and fibrillar Aβ deposits. Abnormal and swollen axons were largely confined within these structures, being enveloped by microglial and astrocytic processes around both plaque types (Suppl. Fig. 3a,b). Labeling for phosphorylated tau using PS442 and AT8 revealed a strong enrichment of PHFs and NFTs in the vicinity of both dense-core and fibrillar Aβ plaques (Suppl. Fig. 4a) intermingled with microglia (Suppl. Fig. 4c,d) and astrocyte processes (Suppl. Fig. 4b,e), further demonstrating that microglia/astrocyte assemblies structurally define areas of degenerating neuronal processes around Aβ plaques. Together the microglia and astrocytes created a unified structure that we refer to as a reactive glial net (RGN) that is reminiscent of a glial scar but with conserved architecture (Figs 3 and 4).

Surprisingly, RGNs showed qualitatively and quantitatively different sub-architectures depending on Aβ plaque type. Dense-core plaque RGNs were consolidated with Iba1+ microglia forming a discrete inner membranous capsule around the core of the plaque followed by an outer shell of reactive astrocytes (Fig. 3a,e). 3D analysis revealed microglia with simplified and amoeboid morphology concentrated at a distance ~20–30 μm of the plaque center. Reactive astrocyte cell bodies were distributed to ~30–50 μm from the plaque, forming a corona around the outer perimeter of microglia (Fig. 3a–c). Reactive astrocytes were polarized with processes surrounding aggregated microglia. Astrocyte reactivity was completed ~80 μm from the plaque. The number of activated microglia and reactive astrocytes recruited to dense-core plaque RGNs correlated with estimated plaque volume (Fig. 3d). Analyzing image stacks showed GFAP+ astrocytic processes were largely excluded from the microglial cell territory encompassing the dense-core plaque (Fig. 3e).

In contrast, fibrillar plaque RGNs displayed more complex anatomy (Fig. 4). The number of microglia and astrocytes recruited to fibrillar plaque RGNs correlated with plaque volume, with a stronger positive correlation between plaque volume and microglial cell recruitment than seen with dense-core plaque RGNs (Fig. 4d). Approximately twice as many Iba1+ microglia were detected within fibrillar plaque RGNs (Fig. 4e). Despite this, the distance of RGN astrocytes to plaques was preserved for both RGN subtypes (Fig. 4e), further emphasizing the extent of microglial cell invasion to fibrillar plaques territories. Unique to fibrillar plaque RGNs, astrocytes extended processes within the microglial territory and Aβ plaque areas (Fig. 4f). Small processes of microglia were also closely intertwined within the fibrillar plaque area, further demonstrating an intricate relationship between microglia and Aβ fibrils (Fig. 4f).

In contrast to dense-core and fibrillar plaques, RGNs do not form around PHF/NFT aggregates in AD cortical tissue. The quantification of the positioning of astrocytes and microglia relative to PHF/NFT aggregates showed that there was no correlation between Iba1+ cell number and NFT/PHF aggregate volume but a small positive correlation with astrocyte cell number was found (n = 19, r = 0.043 for total Iba1+ cells, r = 0.077 for Iba1+ cells within GFAP shell, and r = 0.281 for GFAP+ cells). We observed that microglia do not cluster around these structures but astrocytes sometimes form a corona (Suppl. Fig. 5). Thus, RGNs only form around Aβ plaques and exhibit specific features according to plaque type.

### RGN assembly demarcates progressive multipartite neuropathology in an AD mouse model

**Figure 5:**
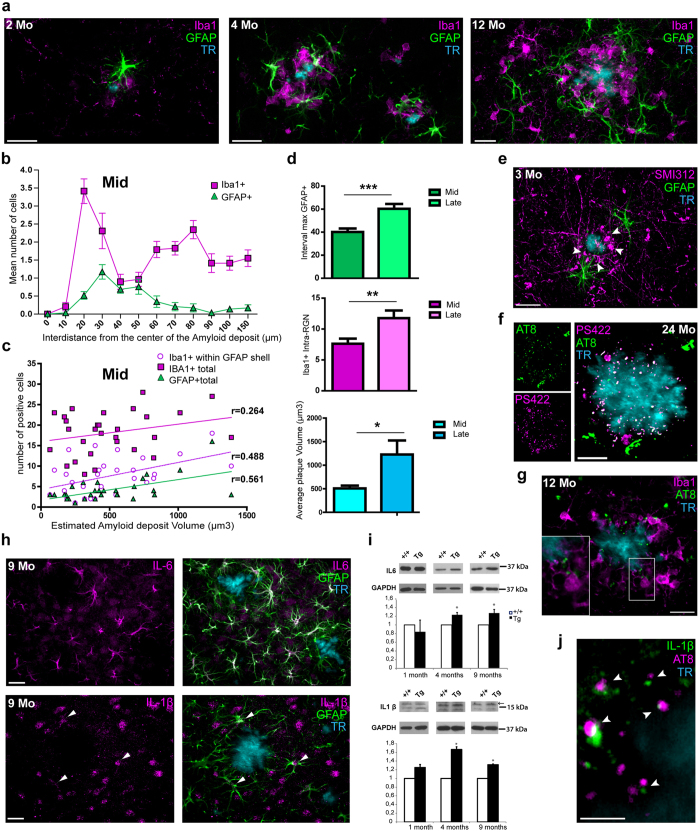
Mouse RGNs in the CRND8 AD model share features with human RGNs and associate with neuronal pathology and Tau granules. (**a**) Time-course for the assembly of RGNs around Aβ deposits. At 2 months, low numbers of activated microglia and reactive astrocytes surround small Aβ deposits. At 4 months, well-constructed RGNs are found. By 12 months, organization of the RGN is degraded and amoeboid microglia and reactive astrocytes can be located distal to RGNs surrounding Aβ deposits. (**b**) 3D analysis of astrocyte and microglia position around Aβ deposits at mid-stages of the disease in the CRND8 model (3–5 months). (**c**) Graph showing the positive correlation between astrocyte and microglial cell number and Aβ deposit volume at mid-stages of the disease (r = 0.264 for total Iba1+ cells, r = 0.488 for Iba1+ cells within GFAP shell, and r = 0.561 for GFAP+ cells; pooled data, 3 to 5 month old mice). (**d**) Comparisons between plaques at mid- (3–5 months) and late-stages (8–9 months) of interval of inter-distance of GFAP+ cells from plaques (F(1, 57) = 14.635; p = 0.0003), numbers of Iba1+ cells within the astrocyte shell of the RGN (F(1, 57) = 7.725; p = 0.0074), and volume of the plaques (F(1, 57) = 5.425; p = 0.0234]. *p < 0.05, **p < 0.01 and ***p < 0.001). (**e**–**g**) Abnormal neuronal processes (e; SMI 312+; magenta) and granules of hyperphosphorylated Tau (**f**,**g**); detected by two different phospho-Tau antibodies PS422 (magenta) and AT8 (green), associated with RGNs. (**h**) Expression of IL-6 (upper panel, magenta) and IL-1β (lower panel, magenta) by GFAP+ astrocytes (green, arrows) in CRND8 mice at 9 months. (**i**) Western blot analysis of overall increases in IL-6 and IL-1β expression (mature form at 17 kDa, arrowhead) in the cortex of transgenic mice at 1, 4 and 9 months (with n = 3 for control and Tg+ at 1 month, n = 4 for control and n = 3 for Tg+ at 4 months, and n=4 for control and n = 4 for Tg+ at 9 months). (**j**) IL-1β clusters (green) are closely juxtaposed to hyperphosphorylated Tau granules (AT8; magenta) in CRND8 mice. Scale bars: 20 μm (**a**,**e**–**h**), 5 μm (**j**).

Mouse models of familial AD (FAD) have played an important role in studying pathways involved in AD. However, a better understanding of how the cellular and molecular alterations seen in mouse models reflect the pathology of AD is needed^33^. Guided by our analysis of human RGNs, we were interested in determining if RGN structures were conserved between AD and mouse model tissue and, if so, the spatio-temporal properties underlying their assembly. One particularly useful FAD mouse model is the CRND8Tg model that expresses the transgene human APP695 cDNA with double mutations at KM670/671/NL (Swedish mutation), along with the V717F (Indiana mutation) under the Syrian hamster prion promoter^34^. CRND8Tg is an early-onset FAD model showing Aβ deposits at 2 months, and Aβ plaques and neuritic pathology by 5 months, thus allowing the full time-course of AD-like disease to be monitored. Interestingly, we detected abundant RGNs at mid-stages (i.e. 4 months; Fig. 5a–c, Suppl. Fig. 6a) in CRND8Tg mice. Time-course analysis in cortical areas showed that starting at 2–3 months (“early-stage”), sparse Iba1+ microglia were found around small Aβ deposits. In some, but not all cases, single GFAP+ astrocytes were in close proximity (Fig. 5a). By 3–4 months (“mid-stage”), the first complete RGNs were present, with microglia encompassing larger Aβ plaques in a rosette conformation and with astrocytes forming an elaborate outer shell-like structure. Microglia extended elaborate processes that circumscribed plaques, similar to microglia around dense-core plaques in AD (Fig. 5a, Suppl. Fig. 6a). Reactive astrocytes surrounding the plaque showed a hypertrophic and highly polarized morphology with their processes creating a complex outer shell of the RGN centered about 40 μm from the Aβ dense-core (Fig. 5d). Microglia and astrocytes displayed a stereotypical arrangement at this time that was influenced by plaque volume (Fig. 5c). By 8 months+ (“late-stage”), an increased recruitment of microglia and astrocytes to RGNs was observed (Fig. 5a,d, Suppl. Fig. 6a,c,d), concomitant with increased plaque volume (n = 30). At this time, the properties of RGNs transformed to incorporate a larger number of activated microglia (Fig. 5d, Suppl. Fig. 6c,d) and encompassed a wider collection of GFAP+ astrocytes within an average of 60 μm inter-distance from the plaque center (Interval Max GFAP+) (Fig. 5d). The appearance of less compact RGNs at late stages in mice resembled the complexity of fibrillar-like plaque RGNs in AD. By 12–24 months, numerous activated microglia and reactive astrocytes adjacent to the RGN structure were frequent (Fig. 5a, Suppl. Fig. 6a) indicating more wide-spread glial reactivity beyond the boundaries of the RGN. Thus, despite differences in the level of overproduction of Aβ plaques in CRND8tg mice, and the temporal progression of AD-like disease, the principle features of RGNs are conserved between AD and an AD mouse model.

### RGNs are toxic and inflammatory microenvironments in AD and a mouse model of AD

We next investigated how RGNs relate to neuronal pathology in the FAD model. Interestingly, abnormal SMI312+ neurites were found within RGNs as early as their formation around 3 months in CRND8Tg mice, increasing in frequency with age (Fig. 5e, Suppl. Fig. 6e,f). MAP2+ dendrites were largely lost from RGNs, with sparse labeling within the GFAP+ astrocyte area (Suppl. Fig. 6g). Surprisingly, neuronal processes containing granules of hyperphosphorylated tau were also found enclosed by RGNs, intermingled between microglia and astrocyte processes (Fig. 5f,g) and confronting abnormal SMI312+ neurites (Suppl. Fig. 6h) as early as 3 months (not shown) and with an increase in number and size over time (Fig. 5 and Suppl. Fig. 6). This was surprising as abnormal hyperphosphorylation and nitration of Tau and insoluble aggregates were only found after 7 months in the CRND8Tg model^35^. At very late stages, phosphorylated tau granules spread outside the RGN with many densities in the surrounding area of plaques often co-distributed with PHF like-structures (Suppl. Fig. 6i,j).

Both microglia and astrocytes express a diversity of proteins whose expression changes with AD^5^ and that can influence neuronal plasticity and health^36^. In particular, the pro-inflammatory cytokines interleukin-6 (IL-6) and interleukin-1β (IL-1β) have been found to be increased in AD^37^,^38^,^39^ and are potent molecules in the exacerbation of tau pathology^40^. To analyze how these cytokines may be related to RGNs, we investigated their expression and localization (Fig. 5h–j, Suppl. Fig. 7). Remarkably, IL-6 and IL-1β were strongly expressed by reactive astrocytes by 9 months, with microglia showing only low-level expression (Fig. 5h–j, Suppl. Fig. 7a–d). IL-6 and IL-1β expression detected in astrocytes at early stages (Suppl. Fig. 7a,c) increased slowly over time and occurred alongside a general increase in astrocyte reactivity (GFAP) and microglial inflammatory markers like major histocompatibility complex II (MHCII) (Suppl. Fig. 6b). IL-1β, in particular, showed an interesting subcellular localization with accumulation in small intracellular clusters located within polarized astrocytic processes labeled with GFAP (Fig. 5h) or glutamine synthetase (GS) (Suppl. Fig. 7d,e) and dispersed with the vicinity of plaques. Remarkably, these IL-1β clusters were closely juxtaposed to aggregates of hyperphosphorylated tau (Fig. 5j, Suppl. Fig. 7f), suggesting a close *in situ* relationship between astrocyte-expressed pro-inflammatory signals and tau hyperphosphorylation. Similar to the human disease where the limbic system is the most vulnerable^41^, we noticed earlier upregulation of IL-6 and IL-1β in astrocytes of the CA1 hippocampus of CRND8Tg mice in the progression of the disease leading to high intensity staining at 9 or 12 months of age (Suppl. Fig. 7e,f, Suppl. Fig. 8c).

**Figure 6:**
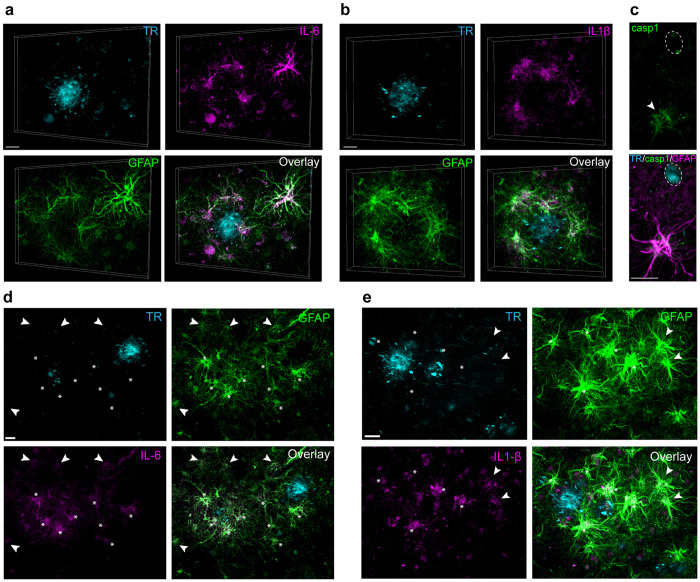
Human RGNs are associated with inflammation in AD. (**a**–**c**) RGN astrocytes in AD tissue express the pro-inflammatory cytokines IL-6 and IL-1β and the IL-1β processing enzyme Caspase 1. (**a**) 3D projection showing IL-6-expressing GFAP+ astrocytes near an Aβ plaque in AD cortex (male, 87 years old). Overlay shows IL-6 (magenta), GFAP (green), and Thiazine red (cyan). (**b**) 3D projection showing IL-1β expression (magenta) in GFAP+ astrocytes (green) around an Thiazine red-labeled Aβ plaque (cyan). (**c**) Reactive astrocytes (magenta) close to Aβ plaques (blue; dotted ellipse) also express the IL-1β processing enzyme Caspase 1 (green; arrowhead) in the same AD patient. (**d**,**e**) Astrocyte are sources of pro-inflammatory cytokines inside and outside RGNs: Examples of astrocytic IL-6 (**d**, magenta) and IL-1β (**e**, magenta) expression inside and outside RGNs with the local astrocyte network (GFAP+, green) in AD cortex. Scale bars: 20 μm (**a,b,d,e**), 10 μm (**c**).

We next determined if the inflammatory features of mouse RGNs corresponded to those in AD. We observed that reactive human astrocytes, and not microglia, were also major source of IL-6 and IL-1β in AD, with highest levels of expression in RGN astrocytes around both dense-core and fibrillar plaques (Fig. 6a,b). Lower intensity staining of IL-6 and IL-1β was observed in neuronal-like structure around plaques and in some instances, both cytokines were localized in isolated reactive astrocytes beyond RGNs, potentially indicating a spread of expression of pro-inflammatory factors into adjacent areas (Fig. 6d,e) Along with IL-6 and IL-1β, caspase 1 (an enzyme that cleaves IL-1β into its mature form)^42^ was accumulated in RGN astrocytes, further demonstrating that astrocytes have detectable levels of molecular components needed for the processing and activation of IL-1β (Fig. 6c).

## Discussion

Here we provide a broadly accessible method that overcomes several limitations that restrict the amount and type of information recovered from human brain tissue in long-term storage. This approach is attractive because of its independence from complex tissue processing and clearing steps. We demonstrate the robustness of the approach by showing how it simultaneously resolves macro- and microscopic features of brain cells in normal and AD tissues that have been stored up to 25 years. Simultaneous imaging of large territories of human brain (mm^2^) and 3D analysis of the relationship of different cellular components is a major advantage of this approach. This relies on the ability of the method to deliver low background autofluorescence, excellent antibody penetration, and uniform labeling in thick slices. At the same time, it also affords the ability to resolve subcellular structures in human brain, from cytoskeletal elements in neurons and glia, to pre- and postsynaptic sites. The usefulness of the approach is exemplified by resolving the complex 3D structure of microglia and astrocytes around Aβ plaques in AD. Furthermore, we show how information obtained from human samples can be used to direct experimentation within an AD mouse model. A. Alzheimer first described dysmorphic microglia and astrocytes around senile plaques in the early 1900’s^6^. Numerous studies have since described this alteration with light and electron microscopy^7^,^8^,^9^, especially in AD mouse models^43^,^44^,^45^. However, the precise 3D organization of these cells and their contribution to the progression of AD still remains unclear^5^. We show that microglia and astrocytes assemble specific RGN structures in AD. Although RGNs show a generalized anatomy with an inner shell of activated and amoeboid microglia enveloped by an outer shell of reactive astrocytes, important differences in RGNs were found that related to Aβ plaque type. At Aβ dense-core plaques, clusters of amoeboid microglia envelop the core Aβ structure and astrocytes processes are intermingled with microglial cell bodies. However, at fibrillar plaques, both microglia and astrocyte processes invade the Aβ plaque area with many close interactions between processes and TR+ fibrils. A higher number of glial cells were recruited to RGNs of fibrillar versus dense-core plaques despite both having similar outer boundaries defined by reactive astrocytes. Interestingly, fibrillar plaques were associated with greater pathology of surrounding neurons^28^, suggesting that an especially dynamic interplay exists between glial cells at fibrillar Aβ deposits. This result corresponds well to the finding that fibrillar plaques are more prevalent in older AD patients with greater disease severity^29^. To further understand the temporal properties of RGNs, we performed a temporal analysis of an AD-like condition in the early onset CRND8Tg mouse model. Initially, RGNs started from small Aβ nucleation sites surrounded by sparse activated microglia and reactive astrocytes. However, by 3–4 months RGNs were more completely formed and closely resembled RGNs around dense-core plaques in AD. In older mice (i.e. 9 months+), RGNs were less consolidated, with some activated microglia and reactive astrocytes located nearby but outside the RGN. The disruption of the RGN organisation is concomitant with a spreading of the pathology in the local environment of the plaques.

Both AD and mouse model RGNs were closely associated with neuronal pathology. In human tissue, PHF and NFT-positive and dysmorphic neurites/neurons were highly concentrated within RGNs. In early and mid-stage CRND8Tg mice, sites of axonal swelling and accumulation of hyperphosphorylated tau granules were first localized within RGNs before spreading locally beyond their boundaries. Interestingly, the more global neuronal pathology corresponded well with an increase in expression of pro-inflammatory cytokines IL-6 and IL-1β in astrocytes, as well as upregulation of generalized markers of reactivity and inflammation including GFAP and MHCII, respectively.

IL-6 and IL-1β are pro-inflammatory cytokines have been found to be increased in serum and cerebrospinal fluid of AD patients^37^,^38^,^39^. Defining the cellular source of these cytokines remains important for designing efficient tools to mitigate the detrimental effect of inflammation in AD. Unexpectedly, we found that reactive astrocytes associated with RGNs were the main producers of both IL-6 and IL-1β in AD and the CRND8Tg mouse model. These data strengthen previous reports of IL-1β expression by astrocytes in other AD mouse models^46^. Interestingly, transcriptome analysis of purified microglia and astrocytes in late stages of the disease in the APPswe/PS1dE9 model suggest that astrocytes engage a robust inflammatory phenotype that rivals and potentially exceeds that found in microglia^47^. The expression of IL-6 and IL-1β in RGN astrocytes is progressive in the CRND8Tg model, suggesting these cells transition to more detrimental roles in later stages. We found that functional and mature IL-1β seem to be trafficked in vesicles/granules along the processes of reactive astrocytes and could potentially be delivered to synapses in adjacent microdomains competent for vesicle release^48^,^49^. Localized cytokine accumulation by astrocytes and their secretion within the RGN could instigate hyper-phosphorylation of tau and contribute to further pathological events like synapse loss and cytoskeletal/protein trafficking disruptions in neurons. This is consistent with the ability of sustained overexpression of IL-1β or IL-6 to significantly increase activity of tau kinases cyclin-dependent kinase 5 (CDK5), glycogen synthase kinase 3β (GSK-3β), and p38 mitogen-activated protein kinase (MAPK)^40^,^50^,^51^. Interestingly, we did not detect significant levels of IL-1β expression in Iba1+ microglia at any stages of the disease in the CRND8Tg and in our collection of human samples. This was surprising as IL-1β production is generally associated with the activation of microglia in numerous pathological contexts^52^,^53^ including AD^5^. It remains to be clarified if microglia produce IL-1β early, but transiently in the disease or modify their activation state accordingly to the influence of reactive astrocytes^54^,^55^,^56^. Microglia play major roles in neurodegenerative processes^17^,^57^,^58^ but could potentially release other cytokines like TNF-α, chemokines, or reactive oxygen species (ROS) to trigger neurotoxic reactions^5^,^59^. It is important in the future to more clearly define the temporal pattern of expression of glial-derived inflammatory cues in the human brain during the progression of AD.

The methodology provided here can be systematically applied to AD brain tissues in long-term storage to more completely and precisely define the spatio-temporal expression profile of neuron and glial proteins involved in AD. Moreover, the universality of the method ensures that it can be used for analyzing tissue from various brain disease settings to improve experimental design in translational brain research.

## Material and Methods

### Human Brain Samples

All experiments involving human tissues were conducted in accordance with the guidelines approved by the Douglas Institute Research Ethics Board. Post-mortem brain samples were obtained from the Douglas-Bell Canada Brain Bank (Douglas Mental Health University Institute, Montréal, QC, Canada). Brain samples were extracted from 5 cases of neuropathologically confirmed sporadic AD of an approximate duration of symptoms of 10–14 years and 5 cases of age-matched controls. On arrival, brains were divided midsagittally: the left hemisphere was cut into thick sections, which were flash-frozen and stored at −80 °C. The right hemisphere was fixed in formalin and examined by a neuropathologist for diagnostic purposes. Hippocampal and temporal cortical samples from all cases were also stained for amyloid plaques and neurofibrillary tangles (NFTs) and assessed for blind Braak staging according to established criteria. The AD cases were selected by the neuropathologist on the basis of a diagnosis according to the CERAD criteria with a Braak amyloid plaque stage of C and a Braak tangle stage between II and VI^23^. In tandem with the neuropathological reports, Braak staging allowed separation of the controls into two groups: (1) Controls, which had no history of dementia and no neuropathological abnormalities, including a complete absence of Aβ plaques and NFTs, and (2) “controls with low AD pathology,” which presented Aβ plaques and tangles with varying degrees, which remained below the threshold for AD diagnosis (see Table 1). Human brain samples used for this study were preserved in formalin until their use.

### AD mouse model

Experiments were approved by the Montreal General Hospital and Douglas Mental Health University Institute Facility Animal Care Committees and followed guidelines of the Canadian Council on Animal Care. CRND8 hemizygous transgenic and age-matched control mice were used for all experiments. In CRND8 mice, a human APP695 transgene with double mutations at KM670/671/NL (Swedish mutation), along with the V717F (Indiana mutation) was inserted into the genome under either the Syrian hamster prion promoter^34^. Wild type and transgenic CRND8 mice from different ages (1 month old up to 24 months old) were anesthetized with isoflurane in a chamber and sacrificed by decapitation. Mouse brains were extracted and fixed overnight in 4% PFA at 4 °C and subsequently washed in phosphate buffered saline (PBS/50 mM potassium phosphate, 150 mM NaCl, pH 7.2).

### Preparation of mouse and human brain slices

Human brain samples and mouse brains were washed in PBS and cryo-preserved in 30% sucrose in PBS for 36 h approximately. Both mouse and human samples were embedded in M-1 embedding matrix (Thermo scientific, USA) or Optimal Cutting Temperature (OCT) compound (Tissue-Tek), and cut into 50 μm to 100 μm -thick slices on a sliding freezing microtome (human) or cryostat (mouse) and kept at −20 °C in a cryoprotectant solution containing ethylene glycol (30%), and glycerol (30%) in 0.05 M phosphate buffer (PB, pH 7.4) until processed for immunofluorescence.

### Immunofluorescence

The protocols for immunofluorescence on mouse and human floating sections were similar with the exception that human brain slices in PBS solution were irradiated for 18–24 hrs with a ultraviolet lamp (Ushio, 30 Watt) prior to the first immunolabeling step in order to reduce background autofluorescence. U.V. irradiation can be accomplished using a standing tissue culture hood equipped with U.V. illumination. This step substantially decreases autofluorescence without appreciable loss of specific cellular labeling. 50 μm thick brain slices were rinsed three times for 10 minutes in PBS followed by a 30 minutes permeabilization step with 0.3% Triton-X 100 in PBS. Subsequently, floating sections were incubated 2 hours with blocking solution (0.3% Triton-X 100, 2% horse serum, in PBS), followed by incubation with primary antibodies (Table 2) in blocking solution for 72 hrs at 4 °C on a horizontal shaker. Double and triple concurrent combinations of primary antibodies were used. To overcome the low immunogenicity of some human samples and the lack of penetration of certain antibodies (Table 2), we added one or two additional steps of 72 hrs of incubation with freshly prepared primary antibody solution (replenishment step). Afterwards sections were washed 3 times for 10 min in PBS, and then incubated with fluorescent tagged-secondary antibodies in 0.3% triton-X 100/PBS at room temperature (RT) for 2 hours. Slices were then washed twice for 10 min in 0.1 M PB (pH 7.4) and some samples incubated with a 300 nM solution of 4′,6-diamidino-2-phenylindole (DAPI) to label nuclei (10 min; RT). To stain Aβ plaques and tau fibrils, sections were incubated in a 0.2 μM Thiazine red solution (Sigma Chemicals, St. Louis, MO, USA) solution for 20 min at RT. Sections were then washed two times for 10 min in 0.1 M PB (pH 7.4) prior to mounting on glass slides using ProLong Gold Antifade reagent (Invitrogen).

To demonstrate deep (z-axis) penetration of multiple labels using the approach, 100 μm thick tissue slices were incubated with 3 rounds of primary antibody (2 replenishment steps; each for 72 hrs). This was followed by 2 rounds of secondary antibody (1 replenishment step; 2 hr incubation). No replenishment of Thiazine red solution was necessary.

### Antibodies

Detailed information regarding the primary antibodies used in this study can be found in Table 2. Donkey secondary antibodies (Invitrogen, Molecular probes, Eugene, Oregon, USA and Jackson ImmunoResearch Laboratories, West Grove, PA, USA) including Alexa Fluor 488 and 647 anti-mouse, anti-rabbit, anti-rat, anti-goat and anti-guinea pig were used at 1:300 (Alexa 488) and 1:400 (Alexa 647) dilutions. Controls with omission of primary antibodies were performed and no background due to secondary antibodies was detected with the exception of mouse secondary antibodies that gave unspecific blood vessel labeling on mouse tissue.

### Imaging and analysis

Confocal images were captured on either an Ultraview spinning disk confocal system (PerkinElmer, Wellesley, MA) or an Olympus FV1000 confocal system (Olympus, Tokyo, Japan) for large area imaging. 3D landscapes of human cortices from age-matched controls and AD patients were reconstituted via the 3D mosaic imaging function a FV1000 Olympus imaging system that facilitates stitching of multiple high-resolution and adjacent Z-stacks. Plaque volumes and inter-distances between center of Iba1+ and/or GFAP+ cells and center of plaques were estimated with Imaris 7.6 software (Biplane) using “Surface Rendering” and “Measurement Points” modules. RGN properties were defined by combining data acquired in the AD post-mortem samples described in table 1. The interval max for GFAP signal corresponds to the average inter-distance interval with the plaque center where the maximum of GFAP+ cells have been found. For each plaque, the interval Max GFAP+ served as the distance defining the limit of the RGN. All microglia included within this distance were attributed to the group of Iba1+ microglia within the RGN (Intra-RGN). Correlations between the number of Iba1+ microglia Intra-RGN, total number of Iba1+ or total number of GFAP+ and plaque/aggregate volume were calculated using the analysis of the coefficient of correlation (r). Statistical analyses were performed with StatView 5.0 software (Abacus Concepts, Berkeley, CA, USA.). A one-way analysis of variance (ANOVA) on plaque factor was performed to compare the influence of the subtype of plaque/aggregates on the interval Max GFAP+ and the number of microglia intra-RGN. All ANOVA analyses were followed by a Tukey/Kramer post hoc test when appropriate. A student t test was used to compare Iba1+ intra-RGN and interval Max GFAP+ between mid- and late-stage of AD mice. The level of statistical significance was set at p < 0.05. For Voronoi analysis, microglia somata were marked in maximum intensity projections of Iba1 images. Delaunay-Voronoi tessellation was performed using a Fiji BeanShell script, taking the centroids of marked somata as tessellation seeds^60^. Complete Voronoi cells were then color-coded according to their area, as measured by ImageJ’s particle analyzer. The same script was used to compute nearest neighbor distances between cells.

### Cortical lysate preparation and Western blotting

Western blot analysis was performed on wild type and CRND8 mice of 1, 4 and 9 months of age (3–4 animals per condition per age, see figure legend) to investigate changes in synaptic, inflammatory, and glial cell protein expression. Cortices from wild type and CRND8 mice were dissected and homogenized in RIPA buffer (1% Triton X-100, 1% sodium deoxycholate, 0.1% SDS, 10% glycerol, 20 mM Tris pH 8.0, 150 mM NaCl and 1 mM EDTA supplemented with 1 μg/ml each of leupeptin, aprotinin, pepstatin, 10 mM NaF, 1 mM sodium ortho-vanadate and 1 mM PMSF) using a Dounce homogenizer and lysed on ice for 30 minutes. Lysates were centrifuged at 13,000 rpm for 10 min at 4 °C to pellet cell debris and protein concentration was determined using a BCA assay (Life Technologies, Burlington, Ontario, Canada). Supernatants were diluted with 3X sample buffer, resolved by SDS-PAGE, and analyzed by immunoblotting using antibodies as detailed in the figure legend and Table 2. Differences between samples were assessed using a 1-sample t test.

## Additional Information

**How to cite this article**: Bouvier, D. S. *et al.* High Resolution Dissection of Reactive Glial Nets in Alzheimer’s Disease. *Sci. Rep.* **6**, 24544; doi: 10.1038/srep24544 (2016).

## Supplementary Material

Supplementary Information

## Figures and Tables

**Table 1:** Chart summarizing control and AD post-mortem brain tissue used in this study.

	Case	Age (years)	Gender	Age of first symptoms	Duration of symptoms	PMI (hours)	Year Fixed
CONTROL							
DH-488	C1	86	F	–	–	5.75	1989 (25)
DH-808	C2	80	F	–	–	17.5	1992 (22)
DH-881	C3	85	M	–	–	5.67	1993 (21)
DH-965	C4	88	M	–	–	15.98	1995 (19)
DH-1117	C5	82	F	–	–	32.58	1999 (15)
AD							
DH-1073	C6	85	M	75	10	35.5	1998 (16)
DH-1157	C7	85	F	71	14	24.75	2000 (14)
DH-1352	C8	82	F	72	10	15	2003 (11)
DH-1631	C9	87	M	76	11	10.8	2008 (6)
DH-1725	C10	77	F	68	10	24.3	2010 (4)

**Table 2:** Chart summarizing antibodies used for immunolabeling procedures.

Target	Manufacturer	Reference number	Host species	Mouse tissue IF	Human tissue IF	WB Mouse
GFAP	SYnaptic SYstems	173 004	Guinea Pig	1/500	1/500	–
GFAP	Millipore	MAB360	Mouse	1/500	NW	1/2000
GFAP	Dako	z0334	Rabbit	1/1000	1/1000	–
Iba1	Wako	1919741	Rabbit	1/500	1/500	–
Iba1	Abcam	ab107159	Goat	1/500	NW	–
Glutamine synthetase	Millipore	MAB302	Mouse	1/500	NW	–
MHCII	Abcam	ab8351	Mouse	–	–	1/5000
PSD95	Cell Signaling	75–066	Rabbit	–	1/250*	–
GAPDH	Abcam	ab9484	Mouse	–	–	1/50000
SMI312	Covance	SMI-312R	Mouse	1/250	1/250*	–
Calbindin D-28k	SYnaptic SYstems	214004	Guinea pig	–	1/250*	–
VGLUT1	NeuroMab	75–066	Mouse	–	1/200*	–
IL-6	Biosource	ARC0062	Rabbit	1/200	–	1/4000
IL-6	Millipore	CBL2117	Mouse	–	1/200	–
IL-1β	Abcam	ab9722	Rabbit	1/200	–	–
IL-1β (mature form)	Abcam	ab2105	Rabbit	–	1/200	–
IL-1β	Rockland	210-401-319	Mouse	–	1/200	1/2000
PS422	Invitrogen	44-764G	Rabbit	1/250	1/250	–
AT8	Thermo Scientific	MN1020	Mouse	1/250	1/250	–

–Not tested; NW: not working in our conditions.

*2 rounds of primary antibody used.

IF, Immunofluorescence.

WB, Western blot.
