# Supplementary material for: High Resolution Dissection of Reactive Glial Nets in Alzheimer’s Disease
Source: Sci Rep. 2016 Apr 19;6:24544. doi: 10.1038/srep24544 (PMC4835751; doi:10.1038/srep24544)
Supplement: Supplementary Information [file srep24544-s1.pdf]

High Resolution of Reactive Glial Nets in Alzheimer's Disease

Abbreviated Title: Reactive Glial Nets in Alzheimer's Disease

David S. Bouvier[1], Emma V. Jones[1], Gaël Quesseveur[1], Maria Antonietta Davoli[3], Tiago Alves-Ferreira[1], Rémi Quirion[2], Naguib Mechawar[2], Keith K. Murai[1*]

Supplementary Figures legends:

Supplementary Figure 1. A method to improve the quality and depth of fluorescence immunolabeling of post-mortem brain samples. (a) Flow chart for preparing, processing, labeling, imaging, and analyzing human brain samples recovered from long-term storage. (b) Ultraviolet light exposure prior the immunolabeling reduces autofluorescence levels in long-term fixed human tissue. Example of the decrease of background levels after Iba1 (magenta) and GFAP (green) immunolabeling on control brain samples (female, 82 years old, 15 years storage) when slices were exposed 24h to U.V. light prior to immunolabeling. Note the unspecific GFAP (bracketed area) and Iba1 labeling (arrowheads) in the non-U.V.-treated samples. Sections were from the same human tissue, processed with the exact labeling conditions, and imaged with the same confocal settings. (c) Successive rounds of primary antibody replenishment increases antibody penetration and signal-to-noise in human sections recovered from long-term storage. Example of the difference in intensity and penetration of the pan-neurofilament antibody SMI312 on control brain samples (female, 82 years old, 15 years storage) between one round and two rounds of incubation with primary antibodies. (d) Multiple rounds of primary and secondary antibody incubations enables deep labeling in thick human brain slices. Deep antibody penetration (up to 100µm in the z-axis) can be

achieved in thick human samples (Alzheimer's patient, male, 87 years old, tissue stored for 6 years) through multiple rounds of primary and secondary antibody incubation. See Methods section for details on labeling procedures. (d') Example z-sections at 20µm intervals and corresponding slice view through the compiled z-stack. Scale bars: 50µm (d, d'), 20µm (b, c), 5µm (c, right panels).

Supplementary Figure 2. Human hippocampal section of post-mortem brain AD patient. Maximum projection of CA1 region of the hippocampus (1.6 mm X 0.87 mm and 40 µm thick) from an AD patient brain samples (male, 78 years old) and labeled with an SMI-312 antibody (pan-axonal filaments) and Thiazine-red (TR) that reveal Aβ plaques, PHF, and NFTs. Scale bar: 100µm

Supplementary Figure 3. Macroscopic pathological inclusions in cortical samples of AD patients. High resolution imaging of the three main subgroups of plaques/aggregates detected by TR staining. (a-b). Abnormal neuronal processes are localized in the vicinity of dense-core and fibrillar Aβ plaques intermingled between microglia and astrocyte processes. (a) Confocal images showing Iba1+ cells (green) enveloping abnormal axons (SMI312; magenta) and located in the vicinity of a dense-core Aβ plaque in AD (male, 87 years old). (b) Confocal images showing GFAP+ cells (green) processes enclosing swollen axons (labeled by SMI; magenta) in the vicinity of a fibrillary Aβ plaque in the same AD patient as above. (c) PHF/NFT aggregates (P) are characterized by a dense cluster of PHFs (open arrows) and NFT (filled arrows) detected by two different phospho-Tau antibodies PS422 (green) and AT8 (magenta) and stained with Thiazine Red (cyan) but are devoid of an Aβ plaque (AD patient; male; 87 years old).Scale bars: 20µm (a-c). (d-e) Irregular distribution of microglia in AD quantified by nearest-neighbor

analysis. (d) Frequency histogram and respective Gaussian fits. A mixture of normal distributions characterizes the heterogenous population of Iba1+ cells. (e) Box-and-whisker plot depicting median (horizontal line), mean (+), and limits of the first and third quartiles. Whiskers indicate minimum and maximum values. n=132 control and 172 AD cells. Mann-Whitney U; ***$p < 0.0001$.

Supplementary Figure 4. Neuronal Tau pathology within RGNs. (a) PHFs and NFTs detected by two different phospho-Tau antibodies PS422 (green) and AT8 (magenta) are found enriched around Thiazine red-labeled Aβ plaques (cyan). (b, c) Low magnification confocal images showing the clustering of PHFs and NFTs, labeled with PS422 (b) and AT8 (c) (magenta) around Aβ plaques and encompassed with GFAP+ astrocytes (b) and intermingled with microglia Iba1+ (c) processes (green) in AD (b, female 77 years old; c, male, 87 years old). (d) Images revealing AT8+ structures (hyperphosphorylated Tau) between Iba1+ microglia (magenta) surrounding Thiazine red-labeled fibrillar Aβ plaque (cyan) in AD (male, 87 years old). (e) 3D reconstruction of a confocal Z-stack with reactive astrocytes of an RGN (green, GFAP) enveloping clustered PHFs and NFTs (labeled with PS422, magenta) around a Thiazine red-labeled dense-core plaque in AD cortex (female, 77 years old). Scale bars: 20 μm (a, b, c); 10 μm (d, e).

Supplementary Figure 5. RGNs do not form around PHF/NFT aggregates in AD cortical tissue. (a) Confocal image of a PHF aggregate in the same AD patient showing Thiazine red-labeled PHF/NFT aggregates (cyan) surrounded by GFAP+ reactive astrocytes (green) but without aggregated Iba1+ microglia (magenta). (b) Quantification of the positioning of astrocytes and microglia relative to PHF/NFT aggregates. (c) No correlation between Iba1+ cell number and PHF aggregate volume but a small positive

correlation with astrocyte cell number is found (n=18; r=0.043 for total Iba1+ cells, r=0.077 for Iba1+ cells within GFAP shell, and r=0.281 for GFAP+ cells). (d) Comparisons between Aβ plaque subtypes and PHF/NFT aggregates regarding the numbers of Iba1+ cells within the astrocyte shell ($F(2,72) = 15.26$; $p < 0.0001$) and the interval of inter-distance of GFAP+ cells from plaque/aggregate ($F(2,72) = 0.047$; $p = 0.9537$). **p < 0.01; ***p < 0.001 from dense-core plaques, ###p < 0.001 from fibrillar plaques. Scale bar: 20 μm.

Supplementary Figure 6. The changes in structural properties of RGNs are accompanied by a local spread of neuronal pathologies. (a) 3-dimensional reconstruction of a confocal Z-stack showing GFAP+ reactive astrocytes (green) and Iba1+ microglia (magenta) surrounding a Thiazine red-labeled Aβ plaque (cyan) in an 4 month old CRND8Tg (early) and in an 8 month old CRND8Tg (late). (b) Western blot quantification of overall changes in GFAP and MHCII expression in the cortex of transgenic mice at 1, 4 and 9 months (with n=3 for control and Tg+ at 1 month, n=4 for control and n=3 for Tg+ at 4 months, and n=4 for control and n=4 for Tg+ at 9 months). (c) 3D analysis of astrocyte and microglia position around Aβ deposits at late-stages (8 to 9 months). (d) Graph showing the increased positive correlation between astrocyte and microglial cell number and Aβ deposit volume at late stages (8-9 months) of the disease (r=0.784 for total Iba1+ cells, r=0.655 for Iba1+ cells within GFAP shell, and r=0.598 for GFAP+ cells; pooled data, 8-9 month old mice). (e-f) First abnormal neuronal structures (labeled with SMI312, magenta) are observed after agglomeration of a certain number of amoeboid Iba1+ cells (green) in RGNs. (g) Neuritic organization around amyloid plaques (*, relative position of Aβ plaque) with dendrites labeled with MAP2 (cyan), axons with

SMI312 (magenta) and astrocytes with GFAP (green) in CRND8 mice at 9 month of age. (h) Phophorylated Tau granules (PS422, green) face swollen neurites (SMI312, magenta) in the vicinity of amyloid plaques in CRND8 mice at 12 month of age. (i-j) Tau pathology (labeled with PS422, magenta) spreading outside RGNs (arrows, i) with PHF-like structures detectable (arrows, j) in very late stages of the disease (22 and 24 Mo CRND8) when astrocyte reactivity (GFAP+, green) is severe and widespread. Scale bars: 20μm.

Supplementary Figure 7. Reactive astrocytes of RGNs are an important source of IL-6 and IL-1β in CRND8 mice. (a) Astrocytes of RGNs (GFAP, green) surrounding Aβ plaques (Thiazine Red, cyan) only express low levels of IL-6 (magenta) in early stages of CRND8 mice. (b, c) Iba1+ microglia (magenta) show only low levels of IL-6 (green, b) and IL-1β (green, c) in CRND8 mice. (d) IL-1β (magenta) is localized in the fine processes of glutamine synthetase (GS)-positive astrocytes (green) but are absent of Iba1+ cells (cyan), and form IL-1β clusters (arrows) in the close proximity of Aβ plaques (*) at 9 months. (e) IL-1β (magenta) is localized in the fine processes of glutamine synthetase (GS)-positive astrocytes (green) near Aβ deposits (Thiazine red, blue) in the hippocampus of CRND8 at 9 months. (f) IL-1β clusters (magenta) correspond to aggregates of hyperphosphorylated tau (AT8; green) in the vicinity of Aβ plaques (TR, cyan). Scale bars: 20 μm (a, b, c, e, f), 10μm (d).

Supplementary Figure 8. Cortical astrocytes do not express significant levels of IL-6 and IL-1β in control human samples. (a,b) The level of expression of the two cytokines IL-6 (a, magenta) and IL-1β (b, magenta) are low or absent in astrocytes labeled for GFAP (green) in control cortex (respectively male, 88 years old and female, 82 years old

age-matched to AD samples). (c) Maximum Intensity projections of IL-1β (magenta) immunostaining in cortical and hippocampal regions of the same animal, a CRND8 at 9 months. Individual hippocampal astrocytes appear to express higher levels of IL-1β than the cortical astrocytes as indicated by more complete labeling of individual cells. Scale bars: 20 μm (a, b).

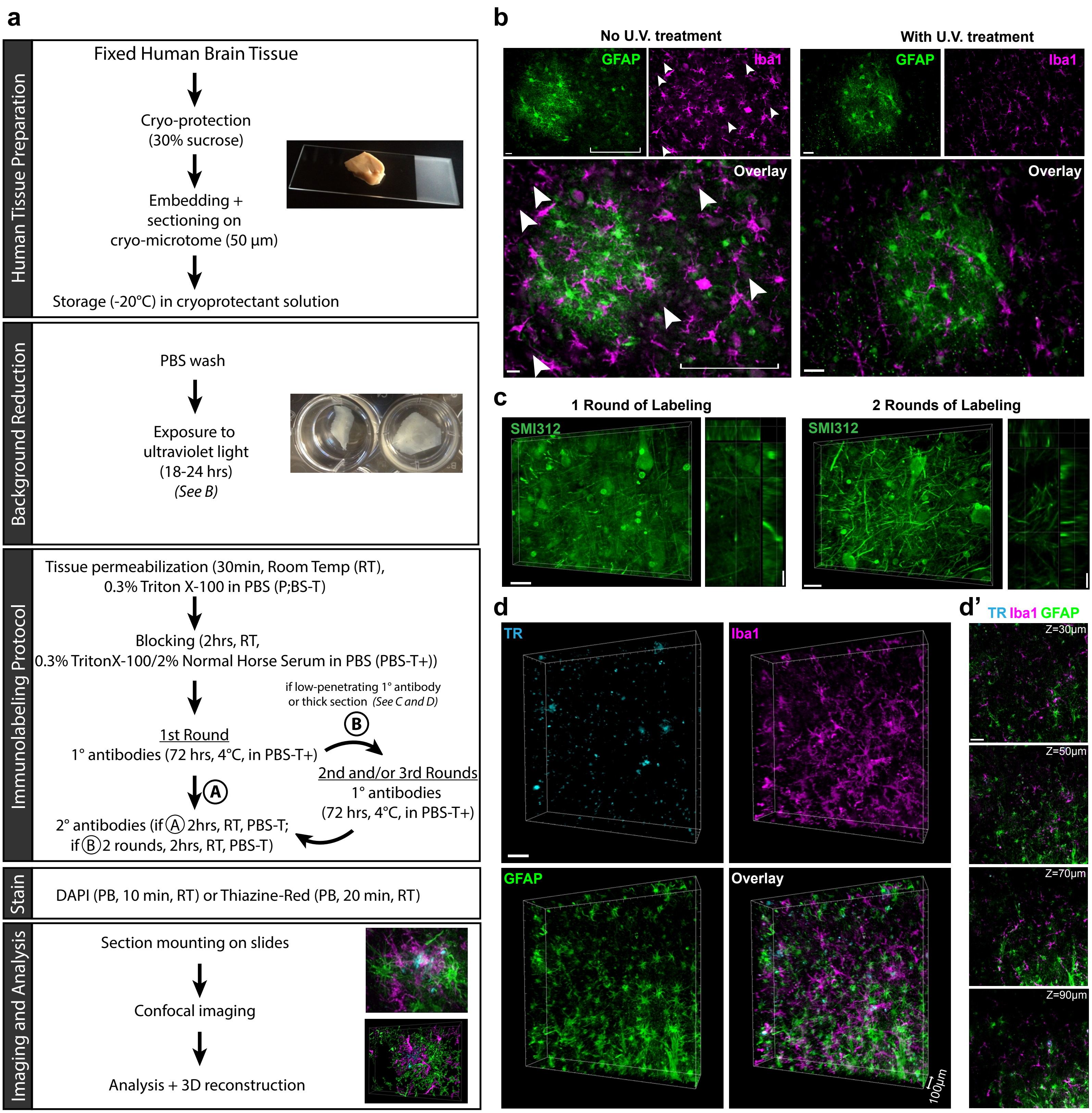

Supplementary Figure 1

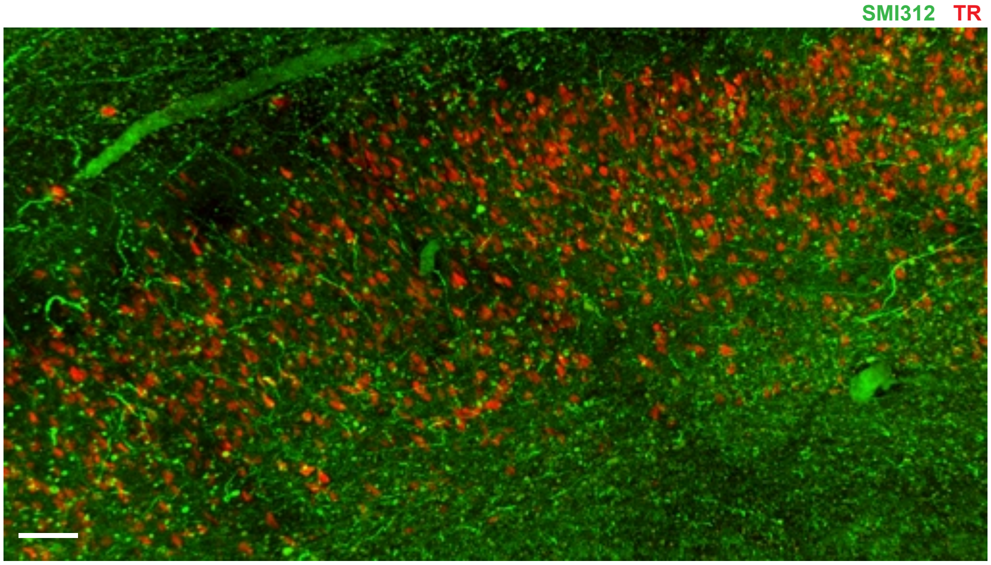

Supplementary Figure 2

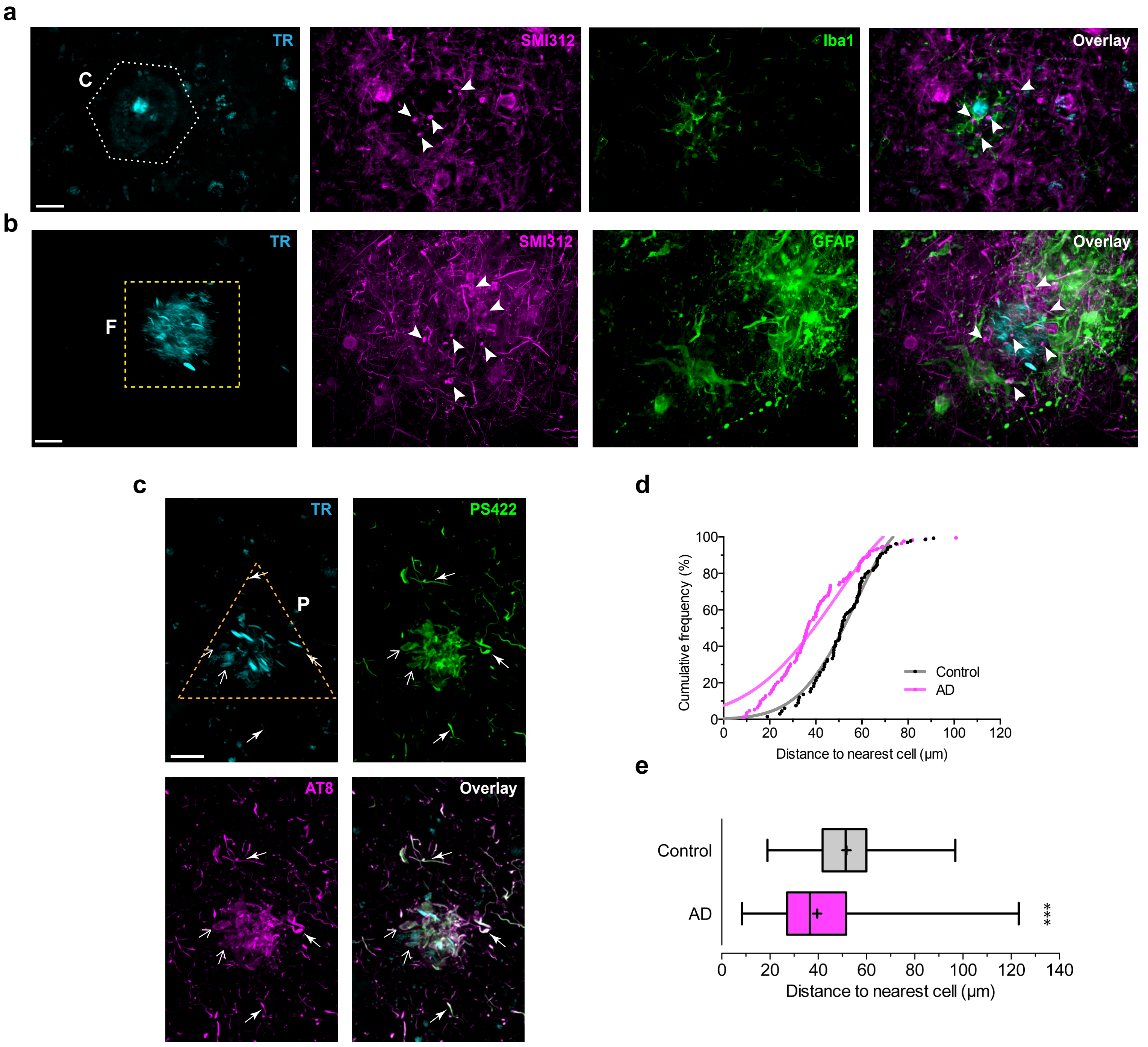

Supplementary Figure 3

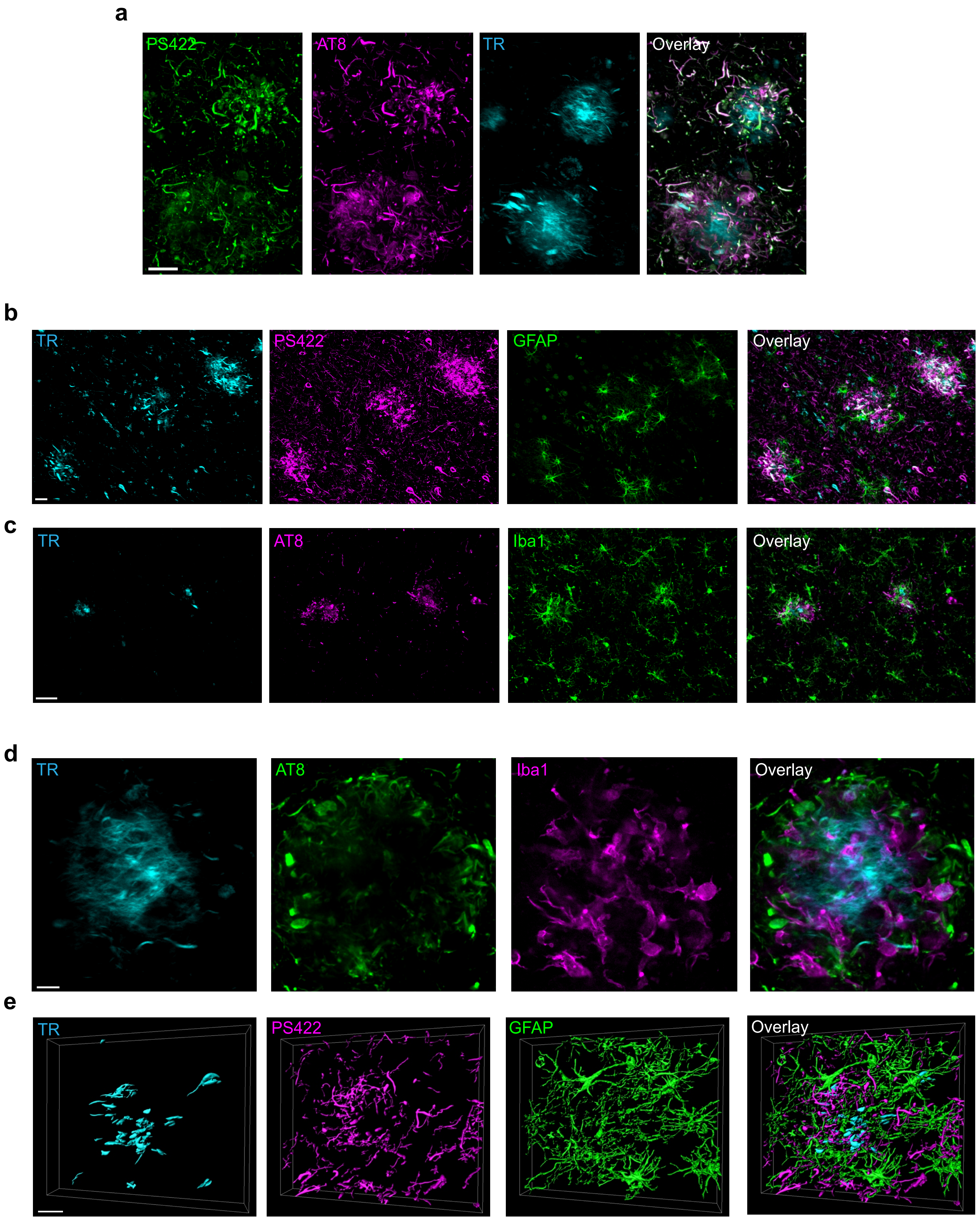

Supplementary Figure 4

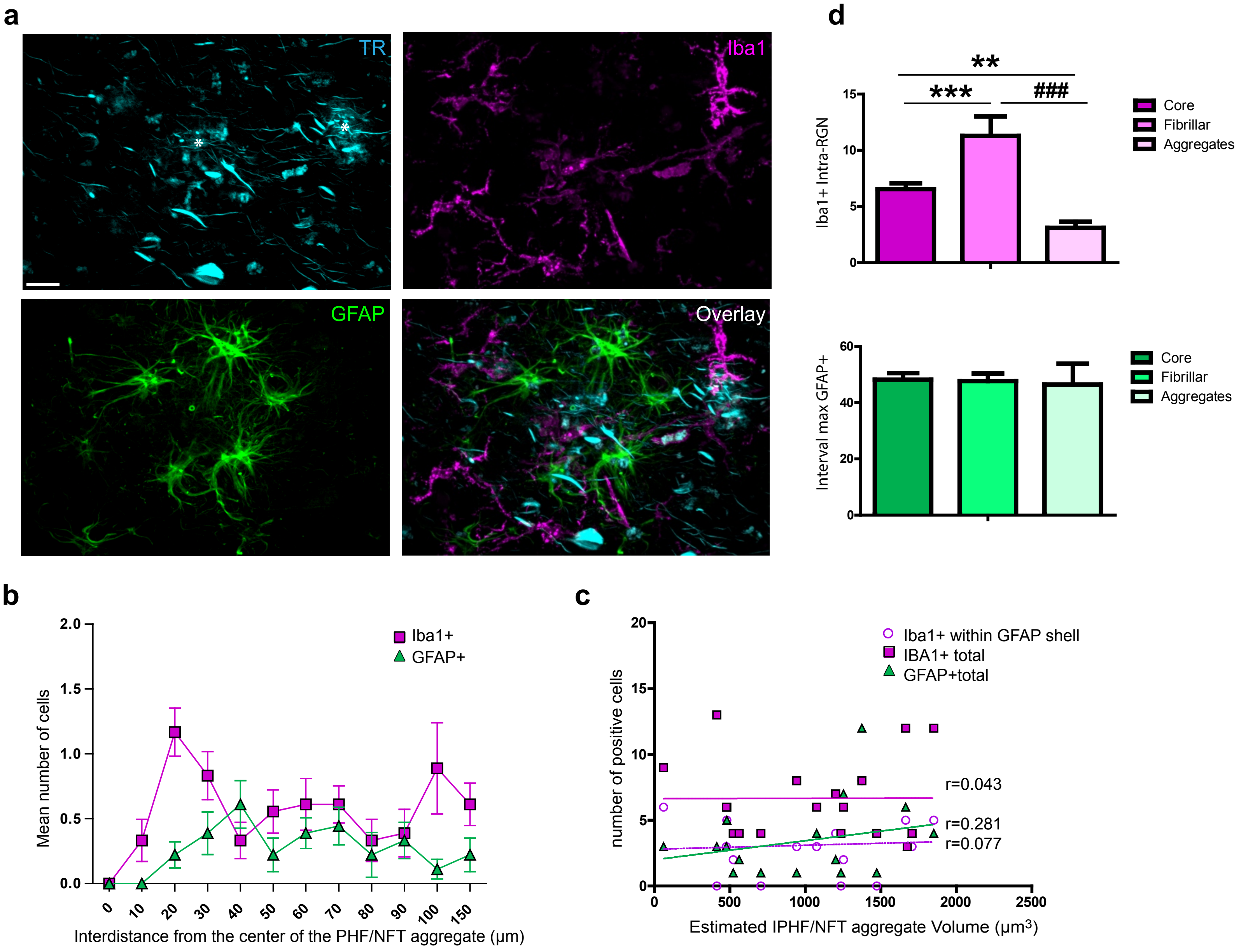

Supplementary Figure 5

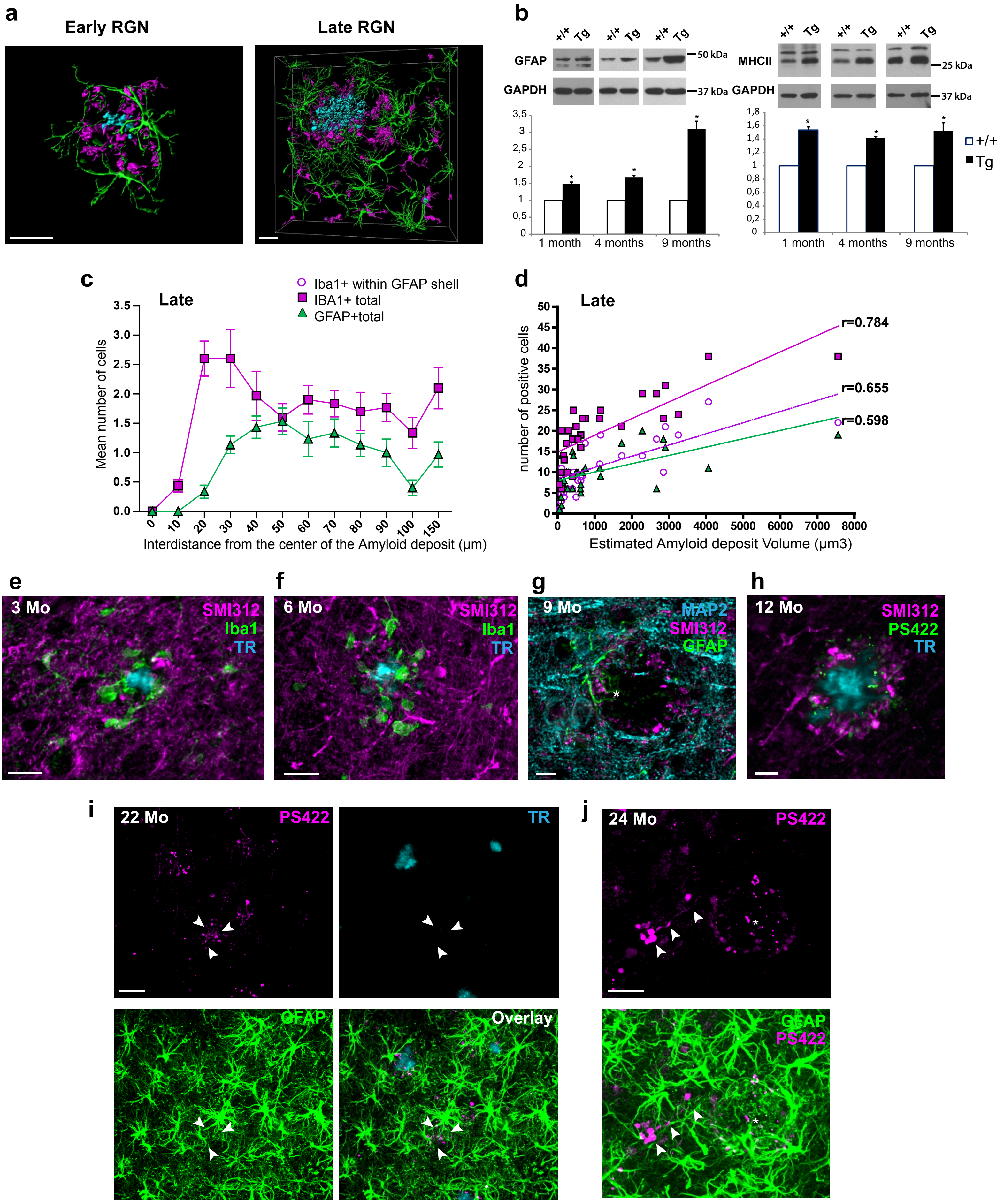

Supplementary Figure 6

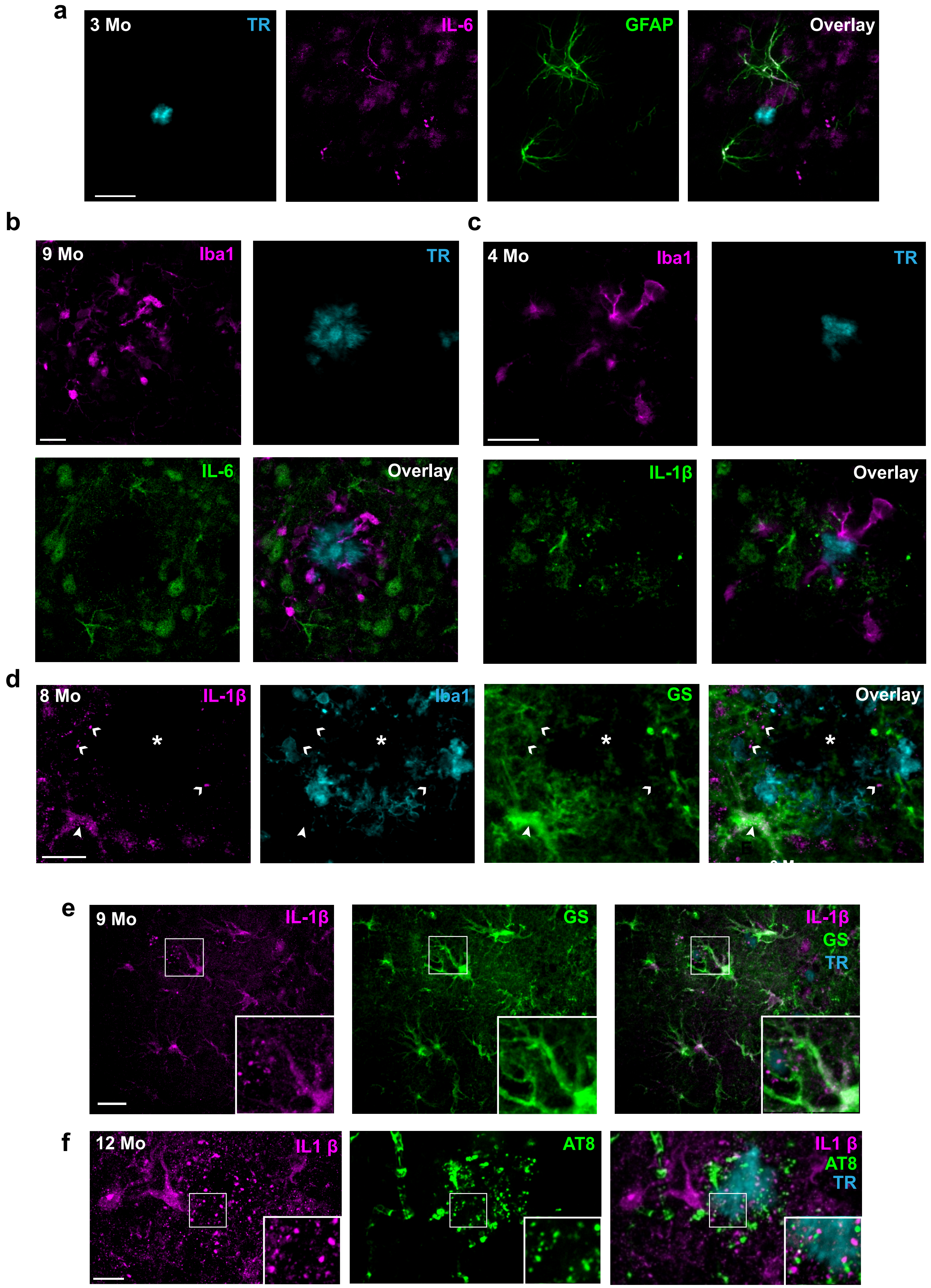

Supplementary Figure 7

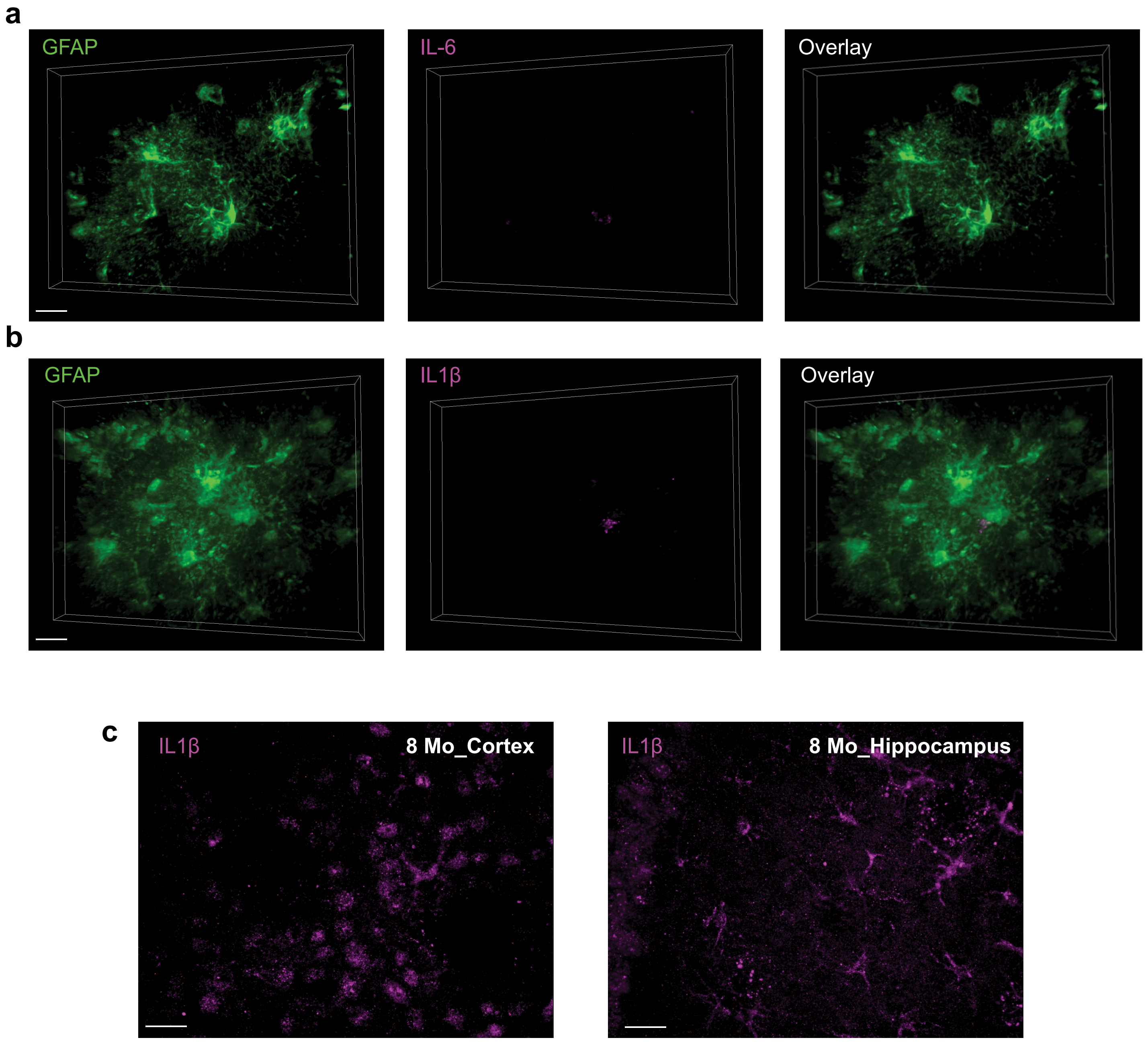

Supplementary Figure 8
